# Effect of odour on prey pattern aversion in jumping spiders

**DOI:** 10.1098/rsos.261295

**Published:** 2026-10-14

**Authors:** Aditi Persad, Laurel B. Lietzenmayer, Lisa A. Taylor

**Affiliations:** Entomology and Nematology Department, University of Florida, Gainesville, FL, USA

**Keywords:** predator, visual, aposematic, aposematism, Hemiptera, priming, hunting, olfactory, warning signal, Salticidae

## Abstract

Many animals defend themselves with toxic chemicals and use multimodal warning displays involving odours and bright colours to advertise their toxicity. Yet, how coloration and odour interact and why they so often evolve together is not well understood. Recent work in jumping spiders has shown that defensive odours from three chemically defended bugs (Order Hemiptera: *Acanthocephala femorata*,* Mormidea* sp. and *Leptoglossus phyllopus*) increase a foraging aversion to red prey. Here, we ask whether these odours also increase avoidance of another visual warning: high-contrast stripes. Jumping spiders (*Habronattus trimaculatus*) were given choices between pattern-manipulated termites (solid white and black-and-white striped) in the presence or absence of a defensive odour from one of the aforementioned bug species. We predicted that any aversion to stripes would be stronger when odour was present. Surprisingly, we found no effect of any bug odour on the spiders’ aversions to stripes. Interestingly, these odours that so strongly and consistently trigger aversion to red do not have the same effect on stripes. This highlights the different ways that odours and coloration can interact (or not) to influence predators, providing insight into the diversity of aposematic displays in nature.

## 1. Introduction

Many aposematic animals have multimodal warning displays that involve bright colour patterns accompanied by noxious odours, and there has been much interest in understanding how and why these components work together to effectively deter predators (reviewed in [1,2]). One idea is that coloration and odour interact, with one component of the signal (typically an odour) ‘priming’ predators to show heightened attention to another component of the signal (typically a conspicuous colour) [2]. To date, most of the work in this field has focused on how noxious odours heighten aversion specifically to long-wavelength warning display colours like yellow and red [3–10]. Interestingly, in these same studies, researchers often find that the same odours that trigger or increase aversions to long-wavelength colours typically have no effect on colours not associated with aposematism (e.g. green, brown, purple). While this effect of odour on long-wavelength colour was initially widely documented using birds as the predators (i.e. all references cited previously), it has, more recently, also been documented with jumping spider predators ([11,12], reviewed in detail below). If the pairing of noxious odours with long-wavelength coloration effectively deters predators across a wide range of sensory systems, from birds to spiders, this may explain why they often evolve together in warning displays.

Yet, when we look at the diversity of aposematic displays in nature, long-wavelength colour is not the only conspicuous visual cue that we see frequently paired with odour. Conspicuous black-and-white stripes are also often paired with defensive odours in many arthropods (see figure 1 and references therein), as well as other animals (e.g. skunks [21]; Eurasion hoopoes [22]; garter snakes [23]). This raises the possibility that odour and striped patterns may work together in similar ways as odour and long-wavelength colours, with odours also triggering aversions to stripes, but evidence testing this idea with any predator is lacking.

**Figure 1.**
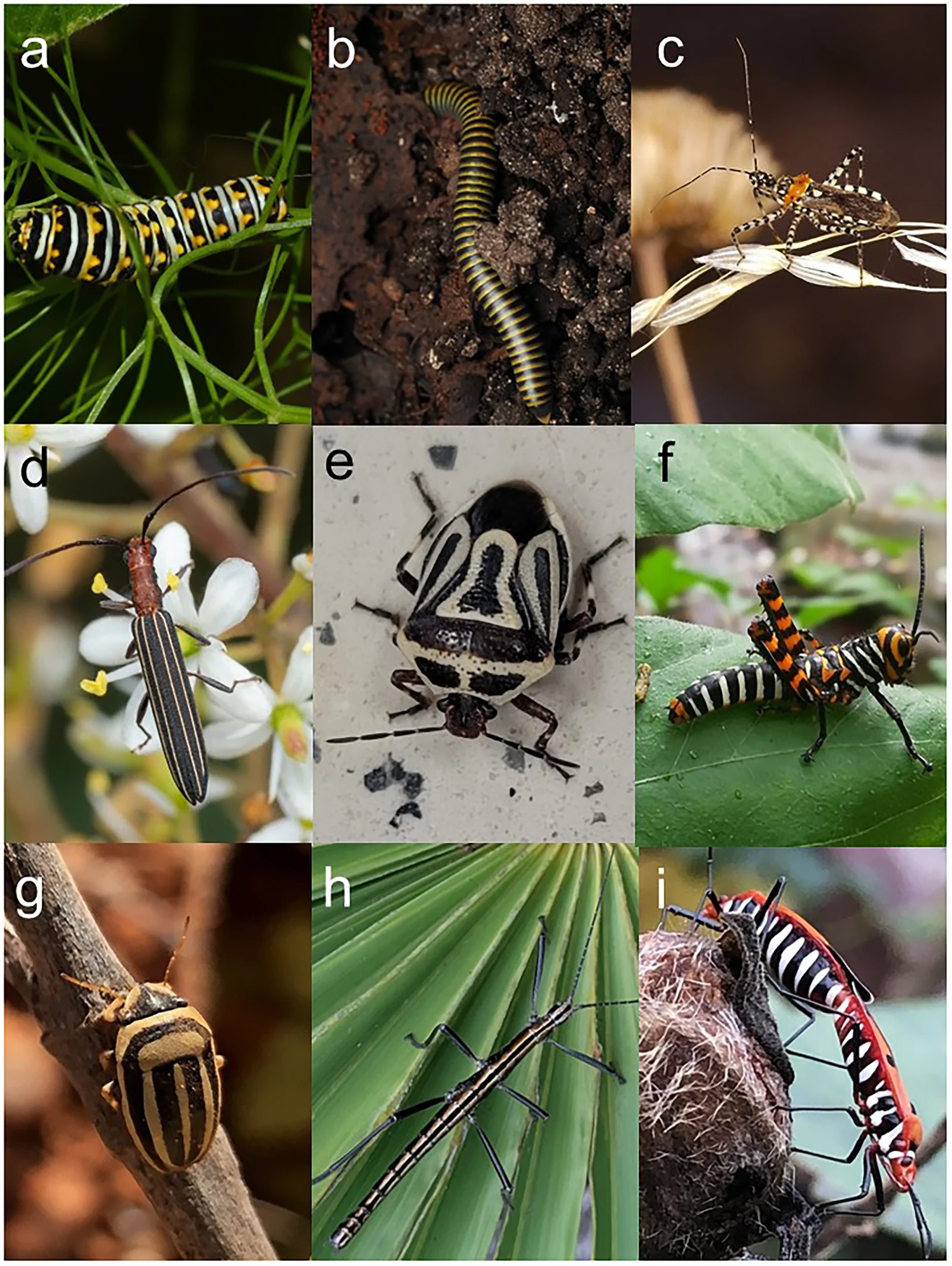
iNaturalist images showing a variety of arthropods that pair high-contrast black-and-white stripes with defensive odours. (a) Swallowtail caterpillar (see [13]), (b) bumblebee millipede (*Anadenobolus monilicornis*) (see [14]), (c) assassin bug (*Pselliopus zebra*) (see [15]), (d) longhorn beetle in the genus *Syllitus* (see [16]), (e) two-spotted stinkbug (*Perillus bioculatus*) (see [17]), (f) giant red-winged grasshopper (*Tropidacris cristata*) (see [18]), (g) stinkbug (*Ippatha australiensis)* (see [17]), (h) southern two-striped walkingstick (*Anisomorpha buprestoides*) (see [19]) and (i) Indian cotton stainers (*Dysdercus cingulatus*) (see [20]). (Creative Commons iNaturalist images by (a) © Ashley Bosarge (CC-BY-NC), (b) © Lucas Garriga (CC-BY-NC), (c) © Michelle W, (CC-BY), (d) © Matt Tudor (CC-BY-NC), (e) © ayden (CC-BY-NC), (f) © Carlos G Velazco-Macias (CC-BY-NC), (g) © Brandi_Layne (CC-BY-NC), (h) © Hope Abercrombie (CC-BY-NC), (i) © Takaaki Hattori (CC-BY 4.0).)

Jumping spiders (family Salticidae) are voracious visual predators that are well-suited for helping us understand complex warning signals. Their central forward-facing principal eyes have exceptional spatial acuity [24] and, in some species, enhanced long-wavelength colour vision (e.g. *Habronattus* sp. [25]). The secondary eyes on the sides of their head are primarily used to detect movement [26]. Movement detection with the secondary eyes results in spiders orienting their entire body to face the subject with their principal eyes [26]; this behaviour helps us discern what captures their attention. Jumping spiders are abundant worldwide, with nearly 7000 species found on every continent except Antarctica [27,28], and feed on a variety of small arthropod prey [29,30]; as such, they are well-suited to help us understand how arthropod defences work. Moreover, there is growing evidence that jumping spiders use both olfactory and visual cues in the context of foraging [29], and that sometimes certain odours can prime spiders to pay attention to visual cues [11,12,31,32].

The North American jumping spider genus, *Habronattus*, is particularly interesting because they are broad generalists that have been observed feeding on a wide variety of prey in nature (e.g. flies, crickets, leafhoppers, termites, other spiders, etc.; L Taylor 2005–2026, personal observation). As small predators (usually 4–6 mm) inhabiting the leaf litter and vegetation, they are likely to encounter many prey items with stripes and/or chemical defences (e.g. small monarch and swallowtail caterpillars [13], striped treehopper nymphs [33], eggs and nymphs of harlequin bugs [34]). From previous work, we have learned about some of the cues that they use to make foraging decisions (e.g. [11,12,35–39]). Moreover, compared to some other jumping spider taxa, they are particularly sensitive to prey toxins [39], which may explain their attentiveness to the colourful warning displays of prey.

Recent work with *H. trimaculatus* has revealed nuanced interactions between responses to odours and colours when foraging [11,12]. Much like in birds, odours can trigger aversions to long-wavelength colour but with some interesting caveats. Vickers & Taylor [11] gave spiders choice tests between red and black colour-manipulated termites and found that when spiders were exposed to the odour from a chemically defended coreid bug (*Acanthocephala femorata*), they were more likely to avoid the red termites compared to when the odour was absent. Interestingly, this odour only increased aversion to the colour red but not to the colour green. Curious about whether this odour-triggered aversion to red was unique to the odour of *A. femorata*, or a broader pattern elicited by other insect chemical defences, Vickers & Taylor replicated this work, testing the effects of four more insect defensive odours [12]. They found that two more hemipteran odours (*Leptoglossus phyllopus*, *Mormidea pama*) triggered the same aversion to red, but the additional odours tested (the ladybird beetle, *Harmonia axyridis*, and the lubber grasshopper, *Romalea microptera*) had no effect on red aversion. Collectively, these results suggest that the priming of attention to red might be something unique to and widespread across hemipteran odours, at least when the predator is *Habronattus* [12].

In this study, we build on this previous work and ask whether the hemipteran defensive odours that strongly and consistently trigger an aversion to red, also trigger an aversion to another conspicuous, aposematic colour pattern common in nature: high-contrast black-and-white stripes. We gave *H. trimaculatus* jumping spiders choice tests between differently patterned prey (termites that had their back patterns manipulated with small pieces of paper (capes) that were either black-and-white striped or solid white) in the presence or absence of hemipteran defensive odours. We predicted that the spiders would attack striped prey at lower rates in the presence of the defensive odours, compared to when the odour was absent. This study broadens our understanding of how multimodal warning displays function and how certain predators may shape different combinations of noxious odours and colour patterns in prey.

## 2. Methods

### 2.1. Preregistration

We preregistered our study hypotheses, predictions, methods and planned statistical analyses with the Center for Open Science prior to collecting data [40]. The purpose of preregistration is to clearly articulate and differentiate between *a priori* hypothesis testing and post hoc exploration of data, thereby reducing bias and increasing objectivity [41,42]. Preregistration is increasingly being encouraged in the field of behavioural ecology (see [43]). Because all three of our experiments (each using a different hemipteran defensive odour) tested the same hypotheses and predictions, all follow the same approach laid out in our preregistration document.

### 2.2. Study species collection and husbandry

We collected juvenile and adult *Habronattus trimaculatus* (figure 2a) measuring at least 4 mm in body length (from abdomen to carapace, rounded to the nearest mm) from North Central Florida. We housed them individually in clear plastic boxes (10.16 × 10.16 × 12.86 cm) containing a 10 cm long artificial green plant for enrichment [44] and a mesh hole for ventilation (approx. 5.5 cm in diameter). The clear boxes were opaque on one side to prevent spiders from seeing and interacting with one another. We fed each spider approximately their own body mass in juvenile crickets (*Gryllodes sigillatus*) and sprayed their boxes with water on alternate days (i.e. every other day). We tested the spiders between 14 and 28 days after collection. To standardize hunger levels, we tested spiders on their scheduled feeding days (prior to feeding) and skipped the feeding day just prior to that; as such, spiders being tested had not eaten for 3 days. Our rationale for this feeding regime was that spiders would be hungry enough to participate in the choice tests, but not so hungry that they would indiscriminately eat the first prey item that captured their attention. No spiders were tested more than once.

**Figure 2.**
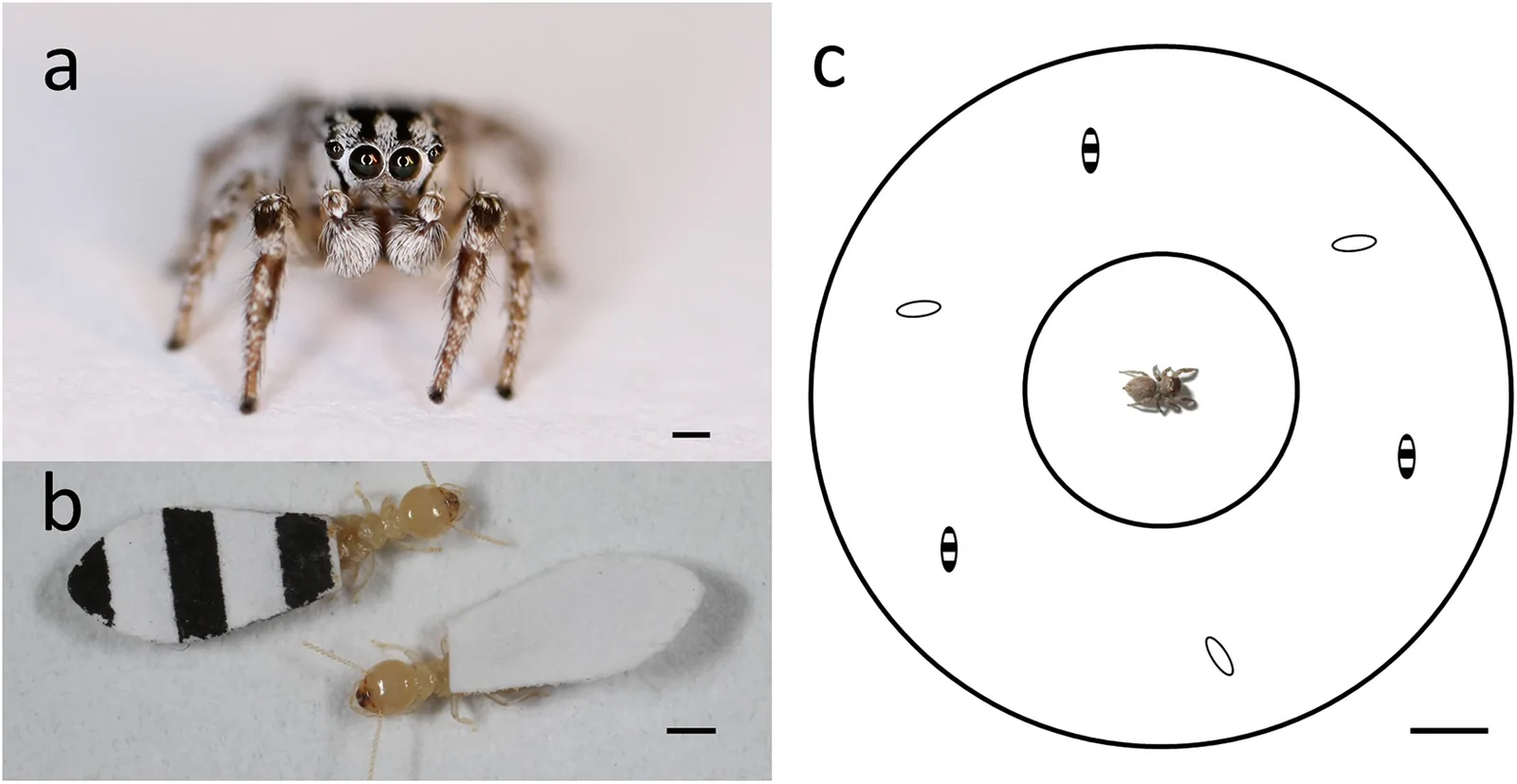
Methods used in prey choice experiments where jumping spiders (*H. trimaculatus*) were presented with arrays of experimentally manipulated termites (*Reticulitermes flavipes*). (a) Adult male *H. trimaculatus* showing the enlarged anterior median eyes that give them their exceptional vision; scale bar represents 0.5 mm. (b) Termites with white and striped paper capes; scale bar represents 1 mm. (c) Illustration of testing arena showing spider in central acclimation chamber before being released to choose among the termites; scale bar represents 1 cm. Termites here are shown equally spaced, but in the actual experiment, the termites were able to move freely around the arena.

Termites (*Reticulitermes flavipes*) were collected from Gainesville, FL and maintained in the lab using buckets and large pieces of rotting wood.

Hemipterans (*A. femorata* (Coreidae), *M. lugens* (Pentatomidae) and *L. phyllopus* (Coreidae)) were collected from North Central Florida and housed in mesh cages with potted tomato (*Solanum lycopersicum*) plants and kitchen sponges moistened with water every other day. Adults of all three of these hemipteran species secrete foul-smelling chemicals that we can smell when the insects are disturbed. These defensive chemicals have been well-characterized in *A. femorata* [45] and *L. phyllopus* [46], with less published on the specific chemicals used for defence in *M. lugens* (but see [47,48], which review the defensive chemicals of pentatomids more broadly). Note that Vickers & Taylor [11,12] used *A. femorata*, *M. pama* and *L. phyllopus*; in our study, we replaced *M. pama* with the closely related *M. lugens* because they were more abundant at our field sites.

The spiders, termites and hemipteran species used in our study are all sympatric and readily abundant around Gainesville, FL, and we often find them in the same habitats. While we have seen the spiders feeding on termites in nature, we have never observed them feeding on the adults of these hemipteran species, probably because they are all relatively large compared with the spiders. Because adult hemipterans typically have different defensive secretions than the small hemipteran nymphs that these spiders might attempt to attack in nature [47,49], the adult hemipteran odours used in our study were likely to be novel to the spiders (see [12]). We chose to use these particular odours to ask the broad question of whether hemipteran defensive odours generally shift the spiders’ responses to stripes.

### 2.3. Striped versus white prey choice tests in the presence/absence of odour

To test how the presence of the hemipteran odours affected *H. trimaculatus*'s propensity to attack striped prey, we ran three experiments, each using a different hemipteran odour. In each experiment, we let the spiders choose among arrays of pattern-manipulated termites in the presence or absence of the odour (see figure 2b,c, see additional details below).

#### 2.3.1. Manipulation of termite pattern

Following previously published methods [35,50–52], we used standard 20 lb. bond white printer paper and an HP LaserJet Pro M402dn printer to create patterned paper capes to adhere to the dorsal surface of the termites (figure 2b). The capes, either striped (white with three horizontal black stripes) or solid white, were adhered to the termite using a small amount of non-toxic school glue (Washable, no-run school glue, Elmer's Products, Westerville, OH, USA) and allowed to dry for 5 minutes. The paper capes were similar in surface area to wings of alate *R. flavipes* (approx. 5 mm long by 2 mm wide; see fig. 3a in [52]); previous pilot data confirmed that these capes did not alter the termite's natural movement rates and that the spiders readily attacked the caped termites [35]. Moreover, previous work confirmed that, at least for *H. trimaculatus*, both white and striped prey were similarly conspicuous when presented on a white paper background (see figure 2b in [35]).

#### 2.3.2. Collection and application of hemipteran odours

We used previously published methods to collect the hemipteran defensive secretions on filter paper so that they could be introduced to the spiders’ choice test arenas (or not, depending on their randomly assigned treatment). To collect the hemipteran odours, we placed an adult hemipteran (*A. femorata*, *M. lugens*, *L. phyllopus)* into a 3.5-cm Petri dish lined with filter paper (cat. no.: 09-803-6D, Fisher Scientific, Pittsburgh, PA, USA) and gently squeezed until they released defensive secretions which were absorbed by the filter paper. These secretions are strong-smelling to humans, allowing us to confirm their release onto the paper. Once we collected the odour for each trial, we replaced the dish lid and then began the trial. Owing to the strength of these odours, we could confirm that they were still present during the trial. While we refer to these secretions as ‘odours’ throughout the manuscript for simplicity, we acknowledge that the spiders might experience these chemicals via some combination of volatile chemoreception (i.e. odour) and contact chemoreception (i.e. taste) directly from the filter paper using chemosensory structures predominantly on the legs (reviewed in [53]).

#### 2.3.3. Choice tests

We placed six caped termites (three solid white and three black-and-white striped) in a 9 cm diameter Petri dish lined with white filter paper, dampened slightly to prevent termite desiccation (figure 2c). We then placed the test spider into an enclosed acclimation chamber (a 3.5 cm clear lidded Petri dish in the centre of the larger termite dish; figure 2c). The floor of the acclimation chamber was lined with filter paper, either with or without hemipteran odour, depending on the spider's randomly assigned treatment. The spiders underwent a 10-minute acclimation period in this setup, in which they could observe the termites from within the acclimation chamber but could not yet interact with them. At the start of this acclimation period, we recorded the colour pattern of the termite (white or striped) that the spider first oriented to. When jumping spiders orient to their prey, they swivel their entire body to align their principal eyes directly with the subject (see [26]). After the 10-minute acclimation period ended, we removed the top of the acclimation chamber and placed a lid on the larger Petri dish, allowing the spider to forage freely among the termites inside of the larger dish. After the spider exited the dish, we again recorded the colour pattern of the first termite they oriented to, the pattern of the first termite attacked and the attack time. Trials ran for 40 minutes or until the spider attacked their first termite.

We recorded trials using overhead video cameras (Sony Handycam HDR-PJ540, Sony, Tokyo, Japan). No spiders or termites were used more than once; spiders that did not attack within the 40-minute trial (*n* = 80 across the three experiments) were not re-tested and were excluded from our confirmatory analyses (see below), as were four trials where the test failed for another reason (i.e. a spider escaped, a cape fell off or the chamber malfunctioned). We ran all trials under SoLux PAR38 3500K 90W full spectrum lights and adjacent to two large windows (1.5 × 0.9 m and 2.8 × 5.0 m) providing natural sunlight. We only tested spiders on sunny or partly sunny days between 10:00 AM and 6:00 PM when these spiders are typically most active (following methods in [11,12]).

### 2.4. Statistical analysis

#### 2.4.1. Confirmatory analyses

To test our focal hypothesis for each of the three different odours, we used one-tailed two-sample tests for equality of proportions to test our main directional prediction that the hemipteran defensive odours would reduce attack rates on stripes. As outlined in our preregistration, we opted to use a one-tailed test here because our main, preregistered *a priori* prediction was explicitly directional and any outcome that did not match this directional prediction (i.e. results in the opposite direction) would result in the same conclusion (i.e. a lack of support for our focal hypothesis; see [54] for the appropriate criteria for using one-tailed tests).

#### 2.4.2. Exploratory analyses

We also ran several exploratory analyses using two-tailed tests; because these were exploratory, they did not meet the criteria above for the appropriate use of one-tailed tests (see [54]).

Because the presence of one type of cue might prime spiders to show heightened attention to a different cue (e.g. [31]), we used likelihood ratio *X*^2^ tests to ask whether the defensive odours (presence vs. absence) affected which type of termite (striped, white) the spider oriented to first.

Because the presence of an odour might affect whether a spider is willing to attack a prey item at all (or delay their willingness to attack), we asked whether the presence/absence of the odour affected whether or not the spider made an attack on any termite (yes, no) using likelihood ratio *X*^2^ tests. We then used two-sample *t*-tests to ask whether the presence of the odour affected the latency to make an attack.

We also ran additional exploratory analyses to examine how these data compare with previous work on *H. trimaculatus* and other jumping spider species interacting with similarly manipulated termites (with black, white and striped capes) in the absence of any hemipteran odours. Previous work found that for some jumping spider species (*Phidippus regius*, *Plexippus paykulli*, *Maevia inclemens*), stripes capture attention and/or reduce attacks on termite prey [35,50,51]. Yet, previous work with *H. trimaculatus* found qualitative trends in the data that hinted at similar patterns but did not meet the threshold for statistical significance, leaving some uncertainty in the conclusions (see [35]). The data collected in this study (i.e. the trials that did not have hemipteran odour present) gave us an opportunity to re-assess patterns of stripe aversion for *H. trimaculatus* with a larger sample size. For this analysis, we subsetted the data and analysed only the trials with the hemipteran odour absent (for a total of 61 trials across the three experiments). We used likelihood ratio *X*^2^ tests to assess whether the spiders were more likely to direct their first orientations towards striped termites compared with white (both within and after leaving the acclimation chamber) and whether they were less likely to attack the striped termites compared with white. We then used a two-sample *t*-test to assess whether they took more time to attack the striped termites compared to the white.

All analyses were performed using JMP 19.0.5. All data from this study are available on Dryad [55].

## 3. Results

### 3.1. Confirmatory analyses

Contrary to our prediction, the presence of the hemipteran defensive odours did not affect which type of prey pattern the spiders attacked in any of the three experiments: (one-tailed two-sample test for equality of proportions: *A. femorata*: *n* = 41, proportion difference = 0.015, *p* = 0.48; *M. lugens*: *n* = 40, proportion difference = −0.10, *p* = 0.73; *L. phyllopus*: *n* = 41, proportion difference = −0.026, *p* = 0.56; figure 3).

**Figure 3.**
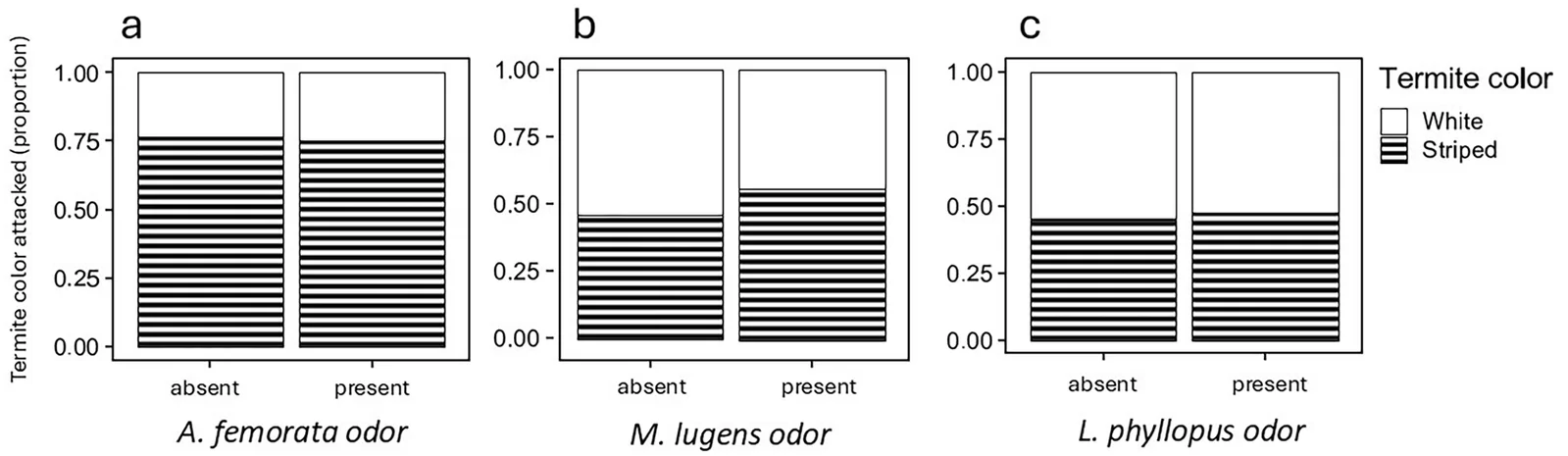
Results of prey choice tests where spiders (*H. trimaculatus*) were given the choice between striped and white caped termites in either the presence or absence of odours from three different chemically defended hemipteran species: (a) *Acanthocephala femorata*, (b) *Mormidea lugens* and (c) *Leptoglossus phyllopus*.

### 3.2. Exploratory analyses

We found no evidence that the presence or absence of the odour affected which type of termite the spiders first oriented to when in the acclimation chamber (*A. femorata*: *X*^2^ = 0.17, *p* = 0.68; *M. lugens*: *X*^2^ = 0.084, *p* = 0.77; *L. phyllopus*: *X*^2^ = 1.97, *p* = 0.16) or the type they first oriented to after they left the acclimation chamber (*A. femorata*: *X*^2^ = 0.29, *p* = 0.59; *M. lugens*: *X*^2^ = 0.70, *p* = 0.40; *L. phyllopus*: *X*^2^ = 0.22, *p* = 0.64).

Interestingly, the effect of the presence of odour on the likelihood of trial participation was different for the different types of odours. For *A. femorata*, the presence of the odour led to significantly more participation (*X*^2^ = 4.45, *p* = 0.035). For *M. lugens*, the presence of the odour did not significantly affect participation, but there was a non-significant trend towards a lower participation rate when the odour was present (*X*^2^ = 3.22, *p* = 0.073). For *L. phyllopus,* there was no effect of odour on participation (*X*^2^ = 0.56, *p* = 0.46). For all three odours, there was no significant effect of the presence of the odour on the latency to make their first attack (*A. femorata*: two-sample *t*-test, mean (± s.e.) latency to attack = 1459.5 seconds (±148.9) when odour absent, 1096.0 seconds (±125.3) when odour present, *t*_39_ = −1.87, *p* = 0.069; *M. lugens*: 1352.4 seconds (±115.8) when odour absent, 1359.4 seconds (±141.9) when odour present, *t*_38_ = 0.038, *p* = 0.97; *L. phyllopus*: 1092.3 seconds (±109.2) when odour absent, 1083.9 seconds (±106.5) when odour present, *t*_39_ = −0.055, *p* = 0.96).

When assessing only the trials where the hemipteran odour was absent (*n* = 61 trials), we found that there were no significant differences in orientations towards striped versus white termites, either when they were in the acclimation chamber (37 striped, 24 white, *X*^2^ = 2.79, *p* = 0.095), or after leaving the acclimation chamber (36 striped, 25 white, likelihood ratio *X*^2^ = 1.99, *p* = 0.16). There was also no difference between the type of termite attacked (33 attacked striped, 28 attacked white, likelihood ratio *X*^2^ = 0.41, *p* = 0.52). In addition, there were no differences in the time it took the spiders to attack striped versus white termites (two-sample *t*-test, mean (± s.e.) time for attack on striped = 1294.5 seconds (±115.8); white = 1300.0 seconds (±125.7), *t*_59_ = 0.032, *p* = 0.97).

## 4. Discussion

In this study, we hypothesized that the presence of hemipteran defensive odours would heighten aversion to striped prey in jumping spiders, in the same way that these odours do for red [11,12]. However, this is not what we found. Across three experiments, using three different hemipteran odours, none had any effect on aversion to stripes. These robust negative results highlight the different ways that odours and colour patterns can interact, or not interact, to influence predators and provide insight into how different odours, colour patterns and other signal components evolve together to create effective multimodal warning displays in nature.

When interpreting results that do not align with our *a priori* predictions, it is important to consider whether these are truly robust negative results (offering evidence against our hypothesis) or, alternatively, whether these odours do alter stripe aversion, but our experiment simply failed to capture it. Our experiments were carefully designed (and preregistered prior to data collection) to be similar to previous work, with the primary exception being the manipulation of prey coloration: black-and-white stripes instead of red. Because of this, we can be confident in concluding that these odours affect *Habronattus* responses to striped prey differently than they do red prey. Publication of robust negative results alleviates the ‘file drawer problem’ where important findings go unpublished [56–58]. Deepening our understanding of the diversity of warning displays in nature requires understanding the strategies that do not work well (e.g. odour and stripes), in addition to the strategies that do work well (e.g. odour and red), for certain predators.

Collectively, our results here alongside previous work [11,12] suggest that, at least for *H. trimaculatus* jumping spiders, odour paired with red might be more effective at deterring predation than odour paired with black-and-white stripes. Why might this be the case? While speculative, there might be something unique about red that makes it particularly salient to *Habronattus*, and therefore more likely to be triggered by hemipteran odours. Numerous studies have examined the perception of long-wavelength colour across jumping spider species (e.g. [59–62]), however most species whose visual systems have been investigated in detail thus far are thought to be ultraviolet-green dichromats; even if they can detect red light, they probably have limited ability to see and discriminate red from other colours [25,63–66]. True long-wavelength colour vision is thought to be limited to only some clades that are spread across the jumping spider phylogeny (e.g. *Habronattus*, *Maratus*, *Stenaelurillus*), although this is an active area of research (see [25,67]). *Habronattus,* in particular, has a unique red filter pigment that gives a subset of their photoreceptors red sensitivity [25]; the resulting trichromatic colour vision may explain *Habronattus*’ heightened attentiveness to red cues. In foraging, they are often averse to red prey [36,38], probably because red is frequently associated with toxicity [68,69], and they can learn to associate red with both unpalatable and palatable prey, depending on their experience [37,70]. In the context of mating, red also seems to be particularly salient to multiple species across the genus [71–73]. It may be that this particular salience of red for *Habronattus* makes it more susceptible to be primed by odour in a way that black-and-white stripes are not. Interestingly, red is also particularly salient to many other animals beyond jumping spiders for reasons that are not fully understood (reviewed in [74]); this might help explain why we see similar patterns of attention to red being primed by odour across animals more broadly (reviewed in §1).

Because this study involved testing spiders for their aversion to stripes in the presence or absence of odour, it gave us an opportunity to revisit a question addressed in a previous study that did not have a clear answer. Gawel *et al.* [35] examined stripe aversion in two species of jumping spiders: *P. regius* and *H. trimaculatus*. They presented spiders with black, white and striped termites and found clear evidence that for *P. regius*, the stripes increased attention but reduced attacks. The patterns for *H. trimaculatus* were less clear. Here, in our subset of trials that had no odour present, we did not find evidence that stripes significantly captured attention more than white (although the trends were in the predicted direction with more orientations directed towards striped prey, see §3). We also found no evidence that stripes reduced attacks, as striped and white termites were attacked at equal rates and in similar amounts of time (with trends in the data leaning opposite expectation, see §3).

Our study adds support to the idea that different species of jumping spiders respond to striped prey in different ways (see discussion in [35]). When trying to understand the selection pressures that predators place on prey, not all predators are the same, even when they are from the same taxonomic group like jumping spiders. With their heightened attention to red, *Habronattus* might attend less to conspicuous achromatic patterns like stripes. By contrast, *Phidippus* appears to be a dichromat, with limited colour vision [25,64]; without the ability to see and discriminate long-wavelength colours like red, they may attend more to achromatic patterns like stripes when deciding what to eat. We should also remember that bold stripes may have other functions in nature beyond just being effective warnings. In many animals, stripes function as disruptive coloration [75] or motion dazzle [76], and, in jumping spiders, bold black lines have even been shown to help jumping spiders recognize each other as other jumping spiders (reviewed by [77]). Our exploratory analyses also highlight the subtle differences that the different types of hemipteran odours can have on a spider's willingness to even participate in an experiment, once again leaving us with additional questions to explore.

Most of our understanding of how and why complex prey defences work comes from birds as the key predators (reviewed in [68]). It is important to understand the various selection pressures placed on prey from different potential predators (e.g. lizards, fish, arthropods, etc.) that vary in their sensory systems and the ways they process information [78–80]. This is particularly important when it comes to smaller arthropod predators because they are exceptionally abundant [81], chronically understudied [82] and have diverse sensory systems and cognitive abilities (e.g. [83,84]). Such understudied predators include jumping spiders but also other visually guided arthropod predators, such as dragonflies [85–87], wasps [88], mantids [89,90], mantidflies [91] and tiger beetles [92]. Exploring responses to complex prey defences in a wider selection of predators can help us determine generalities among all predators versus smaller subsets of them, with the ultimate goal of understanding the diversity of multimodal defence strategies we see in nature.

## Data Availability

All data from this study are available on Dryad [55].
